# Understanding the Barriers to the Integration of Biodegradable Plastics into Healthcare Systems: A Scoping Review Protocol

**DOI:** 10.12688/hrbopenres.14329.1

**Published:** 2026-02-20

**Authors:** David Solola, Andrea Doyle, Keith Rochfort, Aisling Walsh, Killian Walsh, Mary Rose Sweeney

**Affiliations:** 1 Nursing and Midwifery, Royal College of Surgeons in Ireland 26 York Street Campus, Dublin, Leinster, D02 YN77, Ireland; 2 RCSI SIM Lab, Royal College of Surgeons in Ireland 26 York Street Campus, Dublin, Leinster, D02 YN77, Ireland; 3 School of Biotechnology, Dublin City University - Glasnevin Campus, Glasnevin, County Dublin, Ireland; 4 School of Population Health, Royal College of Surgeons in Ireland 26 York Street Campus, Dublin, Leinster, D02 YN77, Ireland; 5 Education, Research and Clinical Support, Royal College of Surgeons in Ireland 26 York Street Campus, Dublin, Leinster, D02 YN77, Ireland; 6 Nursing and Midwifery, Royal College of Surgeons in Ireland 26 York Street Campus, Dublin, Leinster, D02 YN77, Ireland

**Keywords:** Biodegradable Plastics, Healthcare Waste Management, Single-Use Plastics, Sustainable Healthcare, Biopolymers, Barriers, Hospital waste

## Abstract

**Introduction:**

Given its substantially higher-than-average per capita plastic waste and the severe global environmental impacts associated with it, Ireland urgently needs alternatives and effective solutions within its heavily plastic-reliant health sector.

**Objective:**

Within this protocol, a scoping review to identify key barriers to the widespread integration of biodegradable plastics into healthcare is outlined.

**Methods:**

This will be achieved by mapping and synthesising existing literature and the current research landscape regarding the application of these biodegradable plastic materials. The principal objective of this review is to systematically identify barriers, such as the availability of waste disposal facilities, infection control considerations, sterilisation methods, cost, staff and management perspectives, knowledge levels and expertise, as well as other policy frameworks and regulations. This review will be guided by the Arksey and O’Malley methodological frameworks and also report recommendations of Peters et al. This review’s findings are expected to form a foundational knowledge base for health systems, researchers and policymakers.

**Conclusion:**

The obtained evidence will inform the development of robust implementation and research strategies, which will be essential for incorporating biodegradable plastics into healthcare successfully. In turn, this will advance global targets for sustainability, including those relevant to a national context such as Ireland.

## Introduction

An average of 73 kg of plastic is created by each person in Ireland annually, which is double the EU average. ^1^ Plastic waste originating from healthcare institutions contributes greatly to this persisting issue. Across Europe, 36% of all waste coming from hospitals are plastics, with the vast majority being single use. ^2, 3^ A potential solution to this is the introduction of biodegradable alternatives to reduce the large volumes of single-use, fossil fuel plastic consumables that are being disposed of in tonnes currently.

The definition of ‘biodegradable plastic’ used in this review will be the definition provided by Plastics Europe which is: *Plastics degraded by microorganisms into water, carbon dioxide (or methane) and biomass under specified conditions.* ^4^ Today, plastics used in healthcare mainly consist of polypropylene (PP), polyethylene (PE) and polyethylene terephthalate (PET). Polymers such as these have lifespans spanning between hundreds and thousands of years. ^5^

Healthcare risk waste is described as ‘any waste that poses a risk due to its potential infectious nature’. This includes but is not exclusive to, items contaminated with blood, items contaminated with transmissible diseases and items contaminated with bodily fluids such as pus and perinatal fluid. ^6^ A large amount of healthcare risk waste is sent overseas to other European countries such as Germany, France and Belgium. ^7^ Not only is this highly environmentally detrimental, with materials such as polypropylene and polyethylene producing 3.14 kg of carbon dioxide per kg of plastic incinerated. It also comes at a very high cost to the National Health Service (NHS) and the Irish Health Service Executive (HSE). Incineration is by far the most expensive final waste disposal method, costing €1849 per tonne compared to sterilisation which costs €813 per and tonne and landfill which comes in at €130 per tonne. ^8^ Biodegradable plastic consumables would be significantly more environmentally friendly as their final disposal would emit significantly lower amounts of carbon dioxide and would take only between six and twelve weeks to fully biodegrade. Alternative methods to exporting waste for incineration can potentially save the HSE millions per annum.

The HSE released its new Climate Action Strategy in 2023 with plans for it to span until 2050. ^9^ The strategy places the HSE as a significant contributor to the National Climate Action Plan released by the Irish government in 2025, which aims to reduce the country’s emissions on an annual basis. This is a commitment by the HSE to continuing decarbonisation as well as waste reduction. This proposed model contains six priority areas of focus, however areas three and four which are ‘sustainable procurement’ and ‘greener models of healthcare’ respectively, are of most relevance here.

This study examines the barriers to adopting biodegradable plastics in healthcare and explores how they can be used sustainably within the sector. It considers the role of healthcare systems, current and emerging regulations, and economic influences. By reviewing the existing evidence, the study will identify how biodegradable plastics can be integrated into healthcare practices and what strategies may be required to support their effective implementation.

## Methods

The purpose of the review is to explore the barriers to introducing biodegradable plastics into the current healthcare system. Therefore, this review will follow the five key stages outlined by Arksey and O'Malley which are: (1) identifying the research question, (2) identifying the relevant studies, (3) study selection, (4) charting the data as well as, (5) the collation and summarising of results. ^10^ The review will also be guided by recommendations from by Peters et al., specifically with respect to the methods recommended to summarise obtained results as well the methods for the data extraction process as described in further detail below. ^11^

### Review question

The research question for this review was developed using the Population, Concept and Context (PCC) framework. ^11^ In this review, the population refers to the healthcare sector broadly. The concept focuses on the use of biodegradable plastics, including the factors that influence or hinder their adoption. The context relates to healthcare systems, including regulatory, operational and economic environments in which biodegradable plastics may be implemented. Guided by this framework, the research question was refined to:

“What barriers to the adoption of biodegradable plastics in healthcare settings are reported in the literature?”

To address this question, the study will pursue five key objectives:

1. To map how barriers related to material performance are described in the literature.
2. To identify and summarise the regulatory barriers reported.
3. To chart the cost-related barriers discussed in healthcare contexts.
4. To describe barriers linked to human factors, including user acceptance, training and workflow considerations.
5. To explore how the literature addresses barriers related to the final disposal of biodegradable plastics.

### Search strategy

The search strategy for this review involved several steps. First, the selected keywords were combined using Boolean operators across multiple databases with support from RCSI information specialist (KW). PubMed, Embase, Cinahl Ultimate, Cochrane Library and Scopus were used to capture both medical literature and research related to the underlying engineering and scientific aspects of biodegradable plastics. The search strategy applied to each of the databases is presented in the extended data.

To ensure that recent advances in biodegradable plastics and contemporary healthcare waste management policies were included, the search was limited to literature published from 2015 onwards. This timeframe was chosen because 2015 marked the introduction of the United Nations Sustainable Development Goals. Of the seventeen goals, three were particularly relevant to this review: Goal 11 (Sustainable Cities and Communities), Goal 12 (Responsible Consumption and Production) and Goal 13 (Climate Action). ^12^ These goals prompted increased global attention to reducing plastic waste and promoting sustainable materials, including within healthcare.

The inclusion and exclusion criteria shown below in Table 1 will guide the study selection process, and the relevancy of studies for their inclusion/exclusion in the final review.

**Table 1.** The inclusion and exclusion criteria used during study screening for this scoping review.

Inclusion	Exclusion
Studies explicitly discussing biodegradable plastics or biopolymers as an alternative or as a potential alternative to non-biodegradable plastics or other materials.	Studies unrelated to biodegradable plastics or biopolymers.
Biomedical or healthcare-related applications.	Studies in no way related to healthcare or a biomedical application.
If considered a medical device, items discussed would be classified as Grade I or Grade IIa by the European Union Medical Device Coordination Group (MDCG).	If considered a medical device, items discussed would not be classified as Grade I or Grade IIa by the European Union Medical Device Coordination Group (MDCG).
Explicit discussion of barriers, challenges or obstacles to using the mentioned biodegradable plastic within the title, abstract or full text (e.g. cost, infection control, staff acceptance, disposal).	Papers discussing biodegradable plastics without mentioning any barriers or challenges.
Peer-reviewed articles. Qualitative, quantitative, mixed-methods studies and reviews are eligible.	Editorials, opinion pieces, or news articles without substantive evidence or discussion of barriers.
Published from 2015 onwards (to capture modern biodegradable plastic development).	Published before 2015.
Published in English.	Not in English.

The scope of this review was limited to plastics that are considered Class I or Class IIa medical devices, in accordance with the EU guidance, ^13^ or plastics that are not classified as medical devices but are used in the clinical setting. This decision was made as low-risk, non-implantable devices account for a disproportionate share of plastic that enters clinical waste streams. ^14^ Examples of items in these classifications include PPE such as gloves and masks as well as disposable consumables like syringes, tubing and fluid bags (not blood). ^13^ Products in the Class IIb and III categories are usually implantable devices which fall outside the scope of this review as well as the overarching research project. ^13^ These products do not contribute as heavily to waste streams as they are implanted for long periods of time and are sometimes designed to be absorbed by the patient’s body. Limiting this review to Class I and IIa devices therefore enhances the relevance of the findings to ensure this review can support the meaningful reduction of the plastic waste burden from everyday clinical practice.

### Study selection

The results retrieved from the included databases will be exported to Covidence, a web-based platform that facilitates collaborative screening and selection of studies for systematic reviews. Two reviewers (DS and KF) will independently screen the titles and abstracts. Studies that do not meet the inclusion criteria outlined in Table 1 will be excluded at this stage. Any disagreements will be resolved through consultation with a third reviewer (AJD). The remaining studies will then undergo full-text review by the same two independent reviewers, with the third reviewer available to resolve conflicts. The study selection process will be illustrated using a PRISMA flowchart.

### Data extraction

For each study, data will be collected on publication details, including author, year, title, journal, and study type (e.g., review article or original research). Additional information will include the healthcare setting, if applicable (e.g., hospitals or laboratories), and the medical application being investigated, such as single-use devices, medical packaging, or implantable materials e.g. stents.

Physical characteristics of the materials will be extracted, including the type of biodegradable polymer being used, such as polylactic acid (PLA), polyhydroxyalkanoates (PHA), or other biodegradable plastics.

Reported barriers will be extracted and categorised into five main areas:

1. Material properties (e.g., mechanical strength, sterilisation compatibility)
2. Regulatory and policy (e.g., regulatory restrictions)
3. Economic (e.g., cost and funding)
4. Staff-related (e.g., staff attitudes and perceptions)
5. Disposal (e.g., limitations in waste segregation or readily available composting infrastructure)

Two reviewers (DS & KF) will extract data independently from the studies that have been selected in order to ensure consistency and minimise any bias. Any disagreements will be resolved through discussion, with a third reviewer (AJD) who will be consulted if necessary to reach consensus.

### Data synthesis

After data extraction, the findings will be analysed and presented in line with Arksey and O’Malley’s scoping review framework. ^10^ Descriptive summaries will first be used to outline key study characteristics such as publication year, setting, type of biodegradable material investigated as well as type of barrier reported.

Results will be presented in tables as well other graphical mediums such as including histograms and bar charts. This will be accompanied by a narrative synthesis that will describe how selected barriers interact and highlight certain areas where the evidence base is concentrated or limited.

## Pilot search

In order verify and refine the initial search strategy, a pilot search was performed prior to the main screening phase. Results from PubMed were extracted and then randomised using the RANDOM() function in Microsoft Excel. The PubMed IDs (PMIDs) of the first 100 randomized records were then imported into PubData2XL to facilitate the extraction of the article titles and abstracts into a separate.csv file.

The inclusion and exclusion criteria, shown in Table 1 of the review protocol, were rigorously applied to these 100 papers to decide on their relevance and potential inclusion into the final review. Of the 100 papers screened, two papers (2%) met the inclusion criteria. If this yield is extrapolated to the total expected number of unique records to be screened across all databases (n=3097), the theoretical estimated number of included articles would be approximately 62. However, given that PubMed is often considered the most relevant database for this topic, the actual final number of included papers is anticipated to be lower than this initial estimate.

This process allowed for the further refinement and clarification of the inclusion and exclusion criteria and confirmed that the current search strategy successfully identifies sufficient relevant literature to warrant proceeding with the full scoping review.

Each of the selected discussed the use of a biodegradable plastic in a healthcare setting for various biomedical applications. The selected papers were also all published in peer reviewed journals and in English and were published after 2015 as outlined in the inclusion and exclusion criteria in Table 1. Also, each study has a section specifically devoted to the challenges or barriers associated with using biodegradable for their given application. Sadaf et al. discusses several barriers to use of biopolymers in sanitary pads such as optimisation of fibre extraction, surface-modification for moisture management and comfort, biocompatibility testing, and large-scale manufacturability. ^15^ Finally, the review by Mangal et al. (2023) on non-isocyanate polyurethane bioplastics (NIPU) argues that although NIPUs can be derived from more sustainable, renewable feedstocks as well as eliminate toxic isocyanates, several barriers to their adoption exist. ^16^ These include inferior mechanical strength, reduced ability to withstand high temperatures, processing difficulties as well as uncertainties around regulatory approval and long-term stability for medical use.

## Implications for research and practice

The findings of this review will serve to: (1) systematically identify current barriers that are preventing biodegradable plastics from being successfully incorporated into the healthcare system and also (2) to define the pathways through which biodegradable plastics can be successfully integrated into the current global healthcare infrastructure. The findings from this review will act as an evidence base for the informing of policy with regards to biodegradable healthcare plastics, addressing specific areas such as waste disposal methods and economic effects.

The findings from this review will also inform potential future studies in the area of sustainable healthcare materials development and the creation of effective implementation frameworks and strategies which are specifically adapted to different healthcare environments. Furthermore, this review will also identify gaps in the literature regarding biodegradable healthcare plastics and also highlight opportunities to address specific obstacles to making healthcare more sustainable.

This review is designed to provide practical and actionable insights to hospital management as well as policy makers to support evidence-based decision-making with regards to sustainability interventions. Specific interventions may include guiding investment that will go towards the sufficient waste infrastructure necessary for the safe and efficient use of biodegradable plastics in healthcare and also informing the development of comprehensive staff training programs focused on efficient waste segregation and sorting practices.

Ultimately, the outcomes of this review will contribute to advancing sustainable healthcare practices and assisting the sector in aligning its operations with broader global sustainability initiatives, such as the United Nations Sustainable Development Goals.

## Data Availability

The extended data of this protocol is available at https://doi.org/10.17605/OSF.IO/HSVP5. ^17^ This is available on the open repository known as Open Science Framework and have been made available under the Creative Commons Attribution 4.0 International, CC BY 4.0, license. This includes:
• Drafted Search Strategy • PRISMA-ScR Checklist • Data Extraction Guide As this is a scoping review protocol there is no underlying data associated with this article.
