# Supplementary material for: Understanding the Barriers to the Integration of Biodegradable Plastics into Healthcare Systems: A Scoping Review Protocol
Source: HRB Open Res. 2026 Feb 20;9:18. [Version 1] doi: 10.12688/hrbopenres.14329.1 (PMC13620354; doi:10.12688/hrbopenres.14329.1)
Supplement: Peer review history

# Reviewer report: Reviewer response for version 1

**DOI:** 10.21956/hrbopenres.15778.r57889

**Published:** 2026-09-28

**Authors:** Una Fitzgerald

**Affiliations:** 1 University of Galway, Galway, Ireland

The authors have summarised the planned approach for completion of a scoping review on barriers to the integration of biodegradable plastics into healthcare systems. Their citations of peer-reviewed articles and relevant non-peer-reviewed reports clearly justify the need for the scoping review and its potential benefit to those working within or supporting the healthcare sector in Ireland. Phases of planned scoping review are clearly laid out. Inclusion of outputs from a pilot review, based on planned approach, gave useful insights into how to complete next steps.

 Suggest inclusion of statement regarding use or non-use of AI while complete the review. This is especially important considering that this submission sits within 'green' literature.

 In the first sentence of the Introduction, the authors state that “An average of 73 kg of plastic is created by each person in Ireland annually.” This figure dates from 2020 and should be updated to most recent figure. Also, the amount is ‘discarded’ rather than ‘created’.

 Other minor edits suggested include:

 1. For clarity, in the third sentence of the third paragraph of the introduction, suggest inserting ‘in Ireland’, after ‘healthcare risk waste’.

 2. The reference source URL, for reference number 9 (HSE Climate Action Strategy), did not work for this reviewer -but PDF was easily found via search online.

 3. On line 2 of ‘Pilot Search’ section, ‘RANDOM()’ needs correction.

 4. p6, second paragraph – word or phrase is missing after ‘selected’.

 5. Second line of ‘Data Availability’ -substitute ‘has’ for ‘have’, to link to ‘this’ at start of sentence or replace ‘This’ with ‘Data’.

Is the study design appropriate for the research question?

Yes

Is the rationale for, and objectives of, the study clearly described?

Yes

Are sufficient details of the methods provided to allow replication by others?

Yes

Are the datasets clearly presented in a useable and accessible format?

Partly

Reviewer Expertise:

Sustainable lab practices; multiple sclerosis; medical devices; greening medical device reseasrch.

I confirm that I have read this submission and believe that I have an appropriate level of expertise to confirm that it is of an acceptable scientific standard, however I have significant reservations, as outlined above.

# Reviewer report: Reviewer response for version 1

**DOI:** 10.21956/hrbopenres.15778.r57888

**Published:** 2026-09-15

**Authors:** Mercy Okezue

**Affiliations:** 1 University of Michigan, Ann Arbor, USA

Given the importance of replacing single-use plastics with more sustainable alternatives, the authors conducted a scoping review to identify barriers to integrating biodegradable plastics more widely into healthcare. The topic is relevant because of a global trend toward sustainability and reduced plastic waste, given the environmental impacts of plastics. However, the authors can improve the manuscript by following the recommendations I provided.

**Introduction**

1. Items contaminated with pathogens are responsible for transmitting diseases. For clarity, the authors are advised to rephrase “items contaminated with transmissible diseases” in 2 ^nd^ sentence of Paragraph 3.
2. Since the acronyms for polyethylene (PE), polypropylene (PP), etc., were introduced in paragraph 2, line 3, the authors are advised to use these in subsequent parts of the manuscript. For example, see paragraph 3, line 4, and do the same throughout the entire manuscript.
3. For better clarity, consider combining the sentences on lines 4 (“Not only is this highly environmentally detrimental…….”) and 5 (“It also comes at a very high cost to…..”).
4. To improve readability, please delete the conjunction “and” that is in between the words per *and* tonne (“…..which costs €813 per and tonne and landfill…..”) in paragraph 3, line 7.
5. Please provide a citation for the claim made in paragraph 3, line 7: “Biodegradable plastic consumables would be significantly ………. take only between six and twelve weeks to fully biodegrade”
6. One of the most important barriers to reusing/ recycling plastics for medical use is the enforcement of medicines regulatory guidelines. As part of the introduction, the authors are advised to include relevant regulatory barriers (e.g., FDA regulations/policies/guidance)  to recycling plastics for medical use.

**Methods**

  

1. It is not clear why the research question sentence (“What barriers to the adoption ……. reported in the literature?”) after paragraph 2 appears in a different format from the rest of the manuscript. Please fix this and rephrase the question for improved clarity.
2.  Under the Search Strategy section, please write out the full meaning of the acronym “RCSI”, as this is the first time it is used in the manuscript.
3. Also, for clarity, define (KW)
4. To improve reader understanding, the authors are advised to provide a brief overview of the different EU classifications for medical plastics.

Is the study design appropriate for the research question?

Partly

Is the rationale for, and objectives of, the study clearly described?

Yes

Are sufficient details of the methods provided to allow replication by others?

Yes

Are the datasets clearly presented in a useable and accessible format?

Not applicable

Reviewer Expertise:

Medical packaging, early-phase drug development (improving oral drug solubility via nanoparticles), drug product stability studies, and QC laboratory accreditations.

I confirm that I have read this submission and believe that I have an appropriate level of expertise to confirm that it is of an acceptable scientific standard, however I have significant reservations, as outlined above.

# Reviewer report: Reviewer response for version 1

**DOI:** 10.21956/hrbopenres.15778.r53726

**Published:** 2026-03-31

**Authors:** Cassandra Thiel

**Affiliations:** 1 New York University Langone Health, New York, USA

This article summarizes a literature review approach and initial screening for determining barriers to adoption of biodegradable plastics in healthcare settings.

 This is a very important topic. 

 I think the article is fine as-is, but I'm not sure how much would be in the study protocol vs. the final reporting of the resulting study. I think there could be some more information in the introduction - particularly around the landscape of bio-based plastics and what may or may not be included in the definition of biodegradable. Perhaps it could be a method for discerning whether an article under consideration is meeting the described definition (as biodegradable is erroneously applied to lots of bio-based polymers that act exactly like petroleum-based polymers in their end of life). [Example - the four quadrant chart in the middle of this page: https://www.elastomer.kuraray.com/blog/bio-based-polymers/]

 "Compostable" is a term more amenable in the US (California) than biodegradable for plastics, because it can be tested and validated with ASTM standards. This might mean the designation of 'biodegradable' in the study is limited to specific polymers or feedstocks.

 Another note is just a question - why is 2015 the cutoff for articles in the past? Is that a special year in the bioplastics space, or is it just ~10 years ago?

 I think cataloging the barriers may be challenging given where in the larger system they could be occurring - market/economics for the feedstock, manufacturing issues, regulatory hurdles (both in just accepting biopolymer content AND in approving new product designs); that's all upstream. Downstream seems to have some issues - risk of the waste is more determined by blood or other infectious fluids (or chemo, etc.) on the products. There is a lot of confusion or uncertainty around the environmental implications of bioplastics being 'improperly disposed' for their intended disposal. What I mean is, if an item is 'compostable' but only in a proper composting facility, is it worse if it is landfilled and creating methane? What about incineration instead? (We don't necessarily know - there's definitely uncertainty!) Then of course there's use phase issues - do they perform the same as options already on the market?

 Anyway, that aside, the rest of this appears to be a straightforward literature review approach. I'm more familiar with PRISMA methods, but they're very similar.

Is the study design appropriate for the research question?

Yes

Is the rationale for, and objectives of, the study clearly described?

Yes

Are sufficient details of the methods provided to allow replication by others?

Yes

Are the datasets clearly presented in a useable and accessible format?

Yes

Reviewer Expertise:

Life cycle assessment, sustainable healthcare, environmental engineering, systems thinking

I confirm that I have read this submission and believe that I have an appropriate level of expertise to confirm that it is of an acceptable scientific standard.

# Response

**Published:** 2026-08-29

**Authors:** David Solola

**Affiliations:** Nursing and Midwifery, Royal College of Surgeons in Ireland School of Nursing & Midwifery, County Dublin, Ireland

Dear Dr Thiel, 

 Thank you for reviewing this protocol and for your constructive feedback. Please accept my apologies for the delay in replying to your review, as a relatively new PhD scholar I was unfamiliar with the  HRB Open Research process.

 In this scoping review, we aligned our approach with the PRISMA-ScR guidelines. We used the Plastics Europe definition of biodegradable plastics; plastics that are derived from biomass but cannot be biodegraded according to this definition (PET and bio-PE) were not included.

 I wanted to capture more contemporary innovations that are being explored in this sector. To do this, I knew we had to choose a year that was relatively recent as this is a rapidly evolving space, so we aligned the cutoff with the releasing of the UN Sustainable Development Goals. I theorised that this release of the UN SDGs would have caused a surge in research related to sustainability, specifically in healthcare.  

 As you predicted, cataloguing the barriers has been quite challenging, however, six categories have been identified and include Sterilisation Incompatibility,  Inadequate Mechanical Properties and Environmental Concerns. Some of the studies reviewed thus far have discussed the potential environmental effects of biodegradable plastics, specifically when used within the current systems available. I hope the review will also speak to the often-inadequate mechanical properties of these plastics, especially for healthcare consumables.  

 Thank you once again I really appreciate your supportive and thoughtful comments.

 Kind regards,

 David
